# Consequences of animal interactions on their dynamics: emergence of home ranges and territoriality

**DOI:** 10.1186/s40462-014-0020-7

**Published:** 2014-09-03

**Authors:** Luca Giuggioli, V M Kenkre

**Affiliations:** Bristol Centre for Complexity Sciences, Department of Engineering Mathematics and School of Biological Sciences, University of Bristol, Bristol, BS8 1UB UK; Consortium of the Americas for Interdisciplinary Science and Department of Physics and Astronomy, University of New Mexico, Albuquerque, 87131 New Mexico USA

**Keywords:** Animal spacing, Confinement, Movement ecology, Interacting random walks

## Abstract

Animal spacing has important implications for population abundance, species demography and the environment. Mechanisms underlying spatial segregation have their roots in the characteristics of the animals, their mutual interaction and their response, collective as well as individual, to environmental variables. This review describes how the combination of these factors shapes the patterns we observe and presents a practical, usable framework for the analysis of movement data in confined spaces. The basis of the framework is the theory of interacting random walks and the mathematical description of out-of-equilibrium systems. Although our focus is on modelling and interpreting animal home ranges and territories in vertebrates, we believe further studies on invertebrates may also help to answer questions and resolve unanswered puzzles that are still inaccessible to experimental investigation in vertebrate species.

## Introduction

Investigations on the establishment and maintenance of animal spacing patterns and confinement have intrigued researchers for more than a century. Causes and consequences of space use are among the most intensely studied aspects of animal behaviour as is clear from very early reports on territorial behaviour [1-4]. Observations spanning vertebrate and invertebrate phyla have resulted in a consensus that spacing is an innate feature of animal behaviour rooted in basic physiological needs and that it constitutes a fundamental structure through which individuals in social groups live and function (see e.g. [5-15]). Spatial confinement is a crucial feature. Despite its importance, unraveling the mechanisms through which territories, home ranges or other forms of spatial segregation arise, has proved elusive. Spacing patterns can result from the actions of one or more individuals, and serve a variety of functions [16] including the defense of resources and mates (see e.g. [17]), offspring [18,19] and den sites [20]. Other factors are the need to maintain a familiar area to reduce predation risks [21] and a response to intruder pressure [22]. The intermeshing of these different functionalities have made it difficult to define a territory or home range in a way that would embrace all behavioural manifestations of segregation.

A distinction between territories and home ranges put forward by Burt [23] defines a territory as a defended area, that is an area whose exclusive ownership, or at least priority of use, individuals attempt to maintain. A home range, on the other hand, merely represents how an animal has occupied a given region of space; there can be various territorial areas within a home range (see [24] for a more extensive discussion). Burt’s definition represents a conceptual advance in distinguishing territories and home ranges. But it does not provide for a clear delineation of home range boundaries in other contexts. The lack of a rigorous definition for home ranges has caused operational difficulties in determining their size quantitatively. Although major improvements beyond the early estimates of minimum convex polygons [25] have occurred over the years (see e.g. the latest studies in [26-32]), detecting the shape and size of home ranges from the movement of individuals is heavily affected by the sampling frequency as well as the time span to integrate animal locations—daily, seasonally or over the entire lifetime [33,34]. These arbitrary choices determine in different ways whether rarely visited or peripheral areas are included in the size of a home range [35,36]. Current views recognize the intrinsically dynamic nature of a home range, particularly its outer boundaries, and associate its origin to foraging strategies in renewable and patchy resources [37] as well as to an animal’s spatial memory of its environment [16,38].

In the present era of interdisciplinary investigations, it behooves the researcher in any field of science to attempt to profit from insights gained in other fields. The current article subscribes strongly to this philosophy. Characterising random spatial structures has a long tradition in other areas of research such as condensed matter (solid state) physics. Challenges facing solid state researchers are similar to those facing ecologists in this regard [39]. A territory or home range lacks a well-defined periodicity and the resulting structure may depend on the specificity of the underlying substrate, e.g. food distribution, geography of the terrain, vegetation cover, and the initial configuration of the animals in the population. As is the case with observations on amorphous condensed matter aggregates such as glassy and gel systems, ecological experiments require carefully prepared samples and repeated observations to make possible the characterisation of the emerging structures and their comparison to one another. Ensuring repeatability is a great deal more difficult in an ecological setting than in condensed matter systems in the laboratory, mainly because of the deep influence of the heterogeneity of the environment on animal behaviour, for which specific tools such as neutral landscape models have been developed in the last two decades [40-44]. This heterogeneity is partly at the root of intraspecific variability on territory and home range size dependence on resource distribution [14]. In addition, the large number of competing interactions in an animal population—attraction towards resource-rich areas, avoidance of locations visited by a neighbour or a predator, defense of other regions of space, etc.—may make it impossible to reach stationary states that are common place in laboratory systems: ecological systems often tend to remain in long-lived metastable states [45-47]. Metastability is not unknown in condensed matter systems—it occurs in glassy systems (see e.g. [48]) wherein the movement of the individual components can be contingent upon large scale collective rearrangements of the surrounding components. When there is departure from orderly spatial arrangement in a system, special methods of description suited to the treatment of disorder become necessary. When this departure arises from time-dependent dynamics of the interactions between its components, rather than from fixed heterogeneity of the environment, the disorder is called dynamic rather than static [49].

Dynamic disorder has been shown to have profound effects on transport dynamics of quasiparticles in organic crystals. In those systems, crystal vibrations impart to electrons or electronic excitations, the so-called excitons, movement properties quite different from those observed in more rigid solid systems such as inorganic crystals. When the coupling mechanism is strong, the traveling quasiparticle itself becomes heavier and deforms the crystal lattice affecting its own mobility. It thus interacts indirectly with itself trough a dynamic modification of the crystal lattice. These physical phenomena have profound analogy with ecological counterparts resulting in animal spacing patterns. Indirect mechanisms of interaction between animals have been known since 1950s. Pierre-Paul Grassé [50] (see also [51,52]) coined the term *stigmergy* to represent indirect interactions involving the response of insects to changes in the environments made by other insects. That early observation was prompted by mound construction by termites that dropped pellets of chewed earth at various locations until the pellet-dropping activity concentrated at one location to form a column. A much more recent study [53] has also investigated avoidance behaviour with similar lines of reasoning. We find the similarities of the stigmergy concept with dynamic disorder and polaron phenomena [49,54-56] in condensed matter physics to be so obvious that it would be totally inefficient not to utilize in ecology the enormous body of technique and insights that have accumulated in that field.

The integrative nature of animal space use has resulted in the study of spacing patterns blossoming into a truly interdisciplinary endeavour. A need has been felt for a useful paradigm of organism movement [57,58]. Mathematical modelling tools have been increasingly used to analyse movement data (see e.g. [59] and [60]). Experimental devices of lighter weight and higher resolution, such as movement sensors, trackers and data loggers, have continued to be introduced [61,62]. All this has pushed forward the study of animal space use considerably. Motivated by these latest developments, the co-authors of the present article felt that this was the right time for a synthesis on recent investigations on animal spacing patterns.

Given the vast literature on the topic, we also found it appropriate to focus our review on a small subset, in particular on the latest mathematical approaches to study animal home range and territoriality. These have witnessed a great deal of theoretical progress in the last twenty years since the important and recognised work of Lewis, Moorcroft and Murray [63,64]. Our present review will bring our own perspective on the study of spacing patterns which, while it builds on the previous progress, specializes on tools and concepts borrowed from statistical physics and non-equilibrium phenomena. Our interest is less in presenting an overall review of available treatments in the literature and more in providing an account natural to the thinking of the present coauthors.

The rest of the review is organised as follows. In Section “Fundamental considerations for mathematical development” we present our general approach to model animal movement and interactions and introduce some essentials of the mathematical tools we use. In Section “Interactions with the environment: the emergence of home ranges” we discuss home ranges and show in detail how their quantitative extents may be deduced from observations on animal displacements. In Section “Mutual interactions leading to territoriality: the effects of time scale disparity” we focus on territoriality, providing a simplified picture and practical mathematical procedures to study effects of scent-mediated interactions. In Section “Applications to observations in the field” we explain methodologies to extract biological parameters from movement data. Section “Conclusions” contains concluding remarks and thoughts on future directions.

## Fundamental considerations for mathematical development

The problem of free will complicates the description of the motion of animals in comparison to that of inanimate objects. However, observations of the locations of animals exhibit stochastic properties overlaid on consequences of deterministic laws. The stochastic element can be often understood as arising from well defined distributions associated with random walks. *A priori* discussions of a philosophical nature regarding whether this viewpoint is tenable are of little practical use. The test of the validity of such a starting point ultimately lies in a comparison of the observed distribution with the predictions of a random force treatment. Comparisons of this kind have generally shown that in most cases the stochastic method is successful in addressing experimental observations. Our approach in this article is based on such a combination of random walk theory with classical equations of motion constructed with known biological facts and tendencies firmly in mind.

An object located at **x** at time *t* and subjected to Newton’s equations of motion with a force derived from a time-independent potential proportional to *U*(**x**), a random (noise) force *R*(*t*), and an associated damping force proportional to its velocity would obey [65]

(1) $$ \frac{\mathrm{d}^{2}\mathbf{x}}{\mathrm{d}t^{2}}=-\nabla U(\mathbf{x})-\alpha\frac{\mathrm{d}\mathbf{x}}{\mathrm{d}t}+R(t)  $$

where ∇ is a vector indicating differentiation along the axes of the chosen coordinate system and where the mass of the object has been divided out and incorporated in the terms on the right hand side. Under the assumption of high damping, we may drop the inertial term, and making the usual assumptions about the noise (as explained in any text, see e.g. [66]), arrive at the Fokker-Planck equation for the probability density *P*(**x**,*t*) that the object is at **x** at time *t*:

(2) $$ \frac{\partial P(\mathbf{x},t)}{\partial t}=\nabla \cdot \left\{\left[\nabla U(\mathbf{x})\right]P(\mathbf{x},t)\right\}+ D \nabla^{2} P(\mathbf{x},t).   $$

In such a formulation, specification of the particular realization of the random force *R*(*t*) is not required, its properties being reflected in quantities such as the diffusion constant *D* and the fact that what the evolution provides us is probability densities at any given time.

An animal such as a rodent or a fox moving on the terrain may also be regarded as obeying Eq. (2) provided we understand that all decisions by the animal will also be part of the description given by the random force *R*(*t*). Any observable regarding the animal, e.g. its position, the distance between successive steps, or more generally a spatially dependent quantity, is a function of **x**, given by an expression such as *O*(**x**), and its value 〈*O*〉 is predicted within the formalism as

(3) $$ \langle O\rangle=\int \mathrm{d}\mathbf{x}\, O(\mathbf{x})P(\mathbf{x},t).  $$

The above formulation addresses a single animal or many noninteracting animals. Systems studied in this article involve interactions among many animals. In order to extend the description to a group of *N* interacting animals with the *i*th animal at location **x**_*i*_, we focus on the *joint* probability density *P*(**x**_1_,**x**_2_,⋯**x**_*i*_,⋯**x**_*N*_,*t*), and the governing equation takes the form, with the argument of *P* suppressed,

(4) $$\begin{array}{*{20}l} \frac{\partial P}{dt}&=\sum_{i} \nabla_{i} \cdot \left\{\left[\mathbf{F}_{i}(\mathbf{x}_{i})\right]P\right\}+ D_{i} {\nabla_{i}^{2}} P\\ &\quad+\sum_{i} \nabla_{i} \cdot \left[\mathcal{U}(\mathbf{x}_{1},\mathbf{x}_{2},\cdots \mathbf{x}_{i}, \cdots \mathbf{x}_{N})P\right].  \end{array} $$

Equation (4) represents the many-body version of the Fokker-Planck description in Eq. (2), which requires a summation over the animals *i* since each individual may perceive a different force, the one-body interaction *F*_*i*_(*x*_*i*_)=∇*U*_*i*_(*x*_*i*_), due to the spatial heterogeneity in the environment. The second term accounts for the possibility of different diffusion constants for each animal, and the third term with the second *i*-summation describes the many-body interaction among the animals through the interaction term $\mathcal {U}(\mathbf {x}_{1},\mathbf {x}_{2},\cdots \mathbf {x}_{i}, \cdots \mathbf {x}_{N})$.

In systems that one encounters in physics, many-body interactions are almost always taken to be constructed from pairwise pieces. In such a case, the last *i*-summation above would take the form $\sum _{i} \nabla _{i} \cdot \left [\sum _{j\ne i} \mathcal {U}(\mathbf {x}_{i},\mathbf {x}_{j})P\right ]$. In most cases in this article, we will begin with a similar starting point but end up in an effective interaction which is not necessarily pairwise. In describing processes such as transmission of infection in an epidemic, we may further include in the description a label for the state of infection. Eq. (4) represents the general framework with which we will describe macroscopic movement patterns, sometimes termed collective phenomena, that emerge from ‘microscopic’ interactions at the individual level [67].

We will generally take the point of view that the random walks of the animals, equivalently their diffusion, is classical and not anomalous; and that an anomalous nature of diffusion may arise at an effective level because of animal interactions among themselves or with the environment. For instance, the process of foraging might lead to less frequent visits to locations where the food is depleted via earlier visits. The resulting walk may thus possess many of the statistical features of a self-avoiding walk. It is possible that attractive or repulsive interactions with other animals may provide other consequences in the effective motion. While our viewpoint is to look at these as effects rather than features put *a priori* into the system, in some cases we will incorporate the motion as anomalous, right from the beginning of the analysis. In those cases, exemplified by Section “Mutual interactions leading to territoriality: the effects of time scale disparity” below, we will include a memory function in our equations of motion. The diffusion term in (4) may be replaced, in such a case, by

$$D_{i} {\int_{0}^{t}} \mathrm{d}t^{\prime}\,\phi_{a}(t-t^{\prime})\, {\nabla_{i}^{2}} P(\mathbf{x}_{1},\mathbf{x}_{2},\cdots \mathbf{x}_{i}, \cdots \mathbf{x}_{N},t^{\prime}), $$

the suffix *a* on the memory *ϕ* denoting that it represents *anomalous* motion. The anomaly of the movement can be evinced by multiplying the above term by ${x_{i}^{2}}$ and integrating over all possible *x*_*i*_ values. Through integration by parts, in spatially infinite domain, it is straightforward to obtain $2{nD}_{i}{\int _{0}^{t}} dt'\phi _{a}(t')$ where *n* is the number of spatial dimensions. This contribution is equal to 2 *nD*_*i*_ only when *ϕ* is equal to a Dirac delta, i.e. with movement when no memory is present. All other cases give a time dependence characteristic of non-Brownian walks. This feature takes as its basis that all anomalous random walks can be represented as described by appropriate memory functions. Equivalences between continuous random walks and generalized master equations [68,69] and between continuous time random walks and fractional diffusion equations [70] on one hand, and between generalized master equations and fractional diffusion equations [71] on the other have all been demonstrated in the past.

It is also useful sometimes to introduce memory functions in the individual potential term in Eq. (4) to allow for effects such as underdamping so that it reads

$${}{\int_{0}^{t}} \mathrm{d}t^{\prime}\,\phi_{u}(t-t^{\prime}) \nabla_{i} \cdot \left\{\left[\nabla_{i} U_{i}(\mathbf{x})\right]P(\mathbf{x}_{1},\mathbf{x}_{2},\cdots \mathbf{x}_{i}, \cdots \mathbf{x}_{N},t^{\prime})\right\}. $$

However, such is rarely necessary in the description of animal motion.

While memory functions and the associated generalized master equations that employ them [72] are often convenient for analysis, there are situations in which, instead of a description in terms of time-nonlocal kernels in an integro-differential equation, time-dependent transport coefficients such as *D*(*t*) are found to be more natural and useful. A subtlety in such contexts is that it is often then necessary to consider nonlocality in space as well [73]. However, an equivalence between convolution equations with memory and non-convolution but time-dependent equations has been established and a usable prescription provided to go from one formalism to the other [73]. We will see a use of this mathematical viewpoint in Section “Mutual interactions leading to territoriality: the effects of time scale disparity” and Section “Applications to observations in the field”.

We will see that home ranges that emerge from animal interactions with the environment naturally introduce Smoluchowski considerations [74] in the analysis (tethering to an attractive centre) rather than simple diffusion, and that convenient modified pictures arise for the study of territorial behavior stemming from scent-mediated interactions [75]. Considerations such as time scale disparity between processes, an example being the movements of the boundaries of territories relative to the movements of the animals within them [76], will prove to be of crucial importance in our description.

The spatial dependence of the various interaction potentials is gentle in some cases and sharp in others. When the latter is true, the field nature of the potentials (the fact that they are defined at every point in space) may give rise, conveniently, to the dynamics of localized walls or spatial partitions. This can result in mild effects in methodology as when (see Section “Interactions with the environment: the emergence of home ranges” involving repulsive interactions with scent-marking animals, where the analysis is facilitated particularly because of time scale disparity between the wall motion and the animal motion.

Some of the other related tools for the descriptions of interacting random walkers are associated with simple repulsion [77-79], considerations on a discrete lattice [80], when exclusion has a finite range [81], when repulsion occurs within a confined domain [82], in presence of movement in a force field [83,84] and when the walkers have an additional attraction towards each other [85]. When the interactions between animals are attractive rather than repulsive, new phenomena such as flocking or herding arise and have fascinating consequences of their own. We will not deal with them in this article except to point to some exciting recent developments [86-93].

A quintessential problem of animal-animal interactions that has very high human relevance as well is that of the transmission of infection in epidemics. Research on this topic was launched quite early on in the seminal contributions of Anderson and May [94,95] and others [96-98], involving concepts such as mass action, SIR compartmental models, and the basic reproductive rate *R*_0_. Spatial considerations were introduced into these and related areas of study independently by various authors [72,96,99-108] giving the studies a kinetic equation flavor. Missing from some of these studies were confinement features that arise in animal motion from home ranges and yet are clear and compelling in the light of field observations [109,110]. These confinement issues have now been introduced in a natural and mathematically tractable manner in recent investigations [111,112]. We are thus in possession of a usable framework capable of a detailed fundamental study of the transmission of infection in terms of interacting random walks specially under confinement. While of general applicability, the theory has particular relevance to animal movement in zoonotic diseases such as the Hantavirus [113], plague [114,115] and bovine tuberculosis [116,117] as it allows one to understand these disease systems by studying a set of confined random walkers moving on the terrain and transmitting infection on encounter.

## Interactions with the environment: the emergence of home ranges

Animal interactions with the environment will be treated in this section and those among themselves will be covered in the next section. In the absence of all interactions, i.e., when the animals are free random walkers, their probability density may be considered to be a product of individual contributions. The governing equation is thus Eq. (2) with its first term on the right side absent, with the consequence that one has to deal merely with diffusion processes. More complicated individual-level movement could be considered, e.g. correlated or delayed, but for simplicity here we deal only with random wandering and their corresponding diffusive propagators to describe the motion. The probability density of a single animal is essentially identical to the number density of all animals, the only difference being the normalization which is to 1 in the former and to *N*, the total number of animals, in the latter case. Among quantities that can be calculated straightforwardly are the spatial distribution of the animals at any time on the basis of information about it given at the initial time *P*(**x**,0), and derived quantities such as the mean square displacement (MSD) 〈**x**^2^〉 (see e.g. [118,119]). The former is

(5) $$ P(\mathbf{x},t)=\int \mathrm{d}\mathbf{x'} \,\frac{e^{-\frac{\left(\mathbf{x}-\mathbf{x'}\right)^{2}}{4Dt}}}{\left(4\pi D t\right)^{n/2}}P(\mathbf{x'},0),   $$

where *n* is the number of dimensions, and the latter, after the integrations over the terrain are carried out, is known to follow the well-known Einstein relation

(6) $$ \langle\mathbf{x}^{2}\rangle=\int \mathrm{d}\mathbf{x}\,\mathbf{x}^{2}P(\mathbf{x},t)=2nDt,   $$

where the initial value of the MSD has been suppressed on the extreme right side.

A natural development is to augment the basic motion equation by logistic terms to describe sustenance and competition as well as birth and death of the animals, distinguishing, when necessary, between the so-called floaters [120] and resident individuals, and by aggression terms to describe the transmission of infection if it is a matter of concern. Such a detailed framework was constructed and used for the description of the Hantavirus [99,105-107,121,122]. Important to such analysis was the measurement of the quantities employed, in particular the diffusion constant *D*.

With the simple assumption that we are making in most of the present article that the movement of animals is an uncomplicated random walk, i.e., that the governing equation for the motion is a simple diffusion equation, it appears straightforward to measure the diffusion constant *D* from observations of the movements of the animals. The basic theoretical tool is the Einstein relation Eq. (6) between the animal MSD and time. Data considered for this purpose are often, although not always, of the mark-recapture kind, i.e., collected by capturing, tagging, and recapturing the animals in a prescribed (finite) region of space. As will be explained in detail in Section “Applications to observations in the field”, an application to observations on rodents in Panama and New Mexico led to the problem that the MSD, initially indeed linear in *t*, *saturates* for larger *t*, introducing *L*, a saturation length into the description. What is the significance of this length? One way of understanding it is to ascribe it to the fact that the rodents typically move near fixed burrows for reasons of security and food [123-125]. However, another relatively prosaic explanation is also possible. The mark-recapture observations employ a limited region of space where the traps are laid out. This observational feature itself introduces a grid length *G* independently of any characteristics of the animal motion. Either of these two factors could lead to the observed saturation of the MSD. A study of the interplay of the two length scales and a demonstration of how the home ranges of the animals involved can be extracted from the observations, despite their mutual interference, is presented below. For simplicity, we begin our explanation in 1*d*.

The governing equation (2) for the probability density of a single rodent in 1*d* is

(7) $$ \frac{\partial P(x,t)}{\partial t}=\frac{\partial}{\partial x} \left[\frac{\mathrm{d}U(x)}{\mathrm{d} x} P(x,t)\right]+ D \frac{\partial^{2} P(x,t)}{\partial x^{2}},   $$

the center of attraction being the location of the burrow. It is clear that a characteristic spatial extent will emerge in the MSD from features of the potential *U*(*x*), viz., the home range *L* as shown later in Eq. (10) and (12). The mark-recapture method consists of capturing animals at locations *x*_0_ and then recapturing them later at other locations *x*. The aim is to deduce the unknown length *L* with the help of, and despite the interference of, *G*. The latter is provided by the observation technique and known *a priori*. Note that *L* is unknown and is characteristic of the moving rodents. We provide, first a succinct explanation of the procedure given by Kenkre [104], and follow it up with a detailed analysis as given by Giuggioli *et al.* [126].

What makes the problem interesting is that the calculation is for *multiple rodents* each of which has a home burrow located at an unknown and separate location. Let us call these burrow locations *x*_*c*_ and denote by *ρ*(*x*_*c*_) their density. We recall that the steady state distribution of the above probability distribution equation is proportional to $\phantom {\dot {i}\!}e^{-U(x-x_{c})/D}$ and independent of the initial distribution [127]. The MSD calculated from the observations in the steady state is

(8) $$ {}{\fontsize{8.8pt}{9.6pt}\selectfont{\begin{aligned} &\langle \Delta x^{2}\rangle_{\text{ss}}\\ &\quad\!\,=\, \frac{\int_{-\infty}^{+\infty} \mathrm{d}x_{c}\, \rho(x_{c})\int_{-G/2}^{G/2}\mathrm{d}x_{0}\int_{-G/2}^{G/2}\mathrm{d}x\,(x\,-\,x_{0})^{2} e^{-\frac{U(x_{0}-x_{c})+U(x-x_{c})}{D}}}{\int_{-\infty}^{+\infty} \mathrm{d}x_{c}\, \rho(x_{c})\int_{-G/2}^{G/2}\mathrm{d}x_{0}\int_{-G/2}^{G/2}\mathrm{d}x\, e^{-\frac{U(x_{0}-x_{c})+U(x-x_{c})}{D}}}. \end{aligned}}}  $$

Notice that the density of the burrow locations, the probe length *G*, and the home range length *L* which is intrinsic to *U*(*x*) are *all* represented in Eq. (8).

A compact manner of writing the above relation uses [104] Fourier transforms and exploits the relation between moments in real space and derivatives in reciprocal space. If the distribution of the burrow locations *ρ*(*x*_*c*_) is taken to be uniform over the terrain for simplicity, we get

(9) $$ \langle \Delta x^{2}\rangle_{\text{ss}}=- \frac{\int\mathrm{d}k\, \frac{\partial^{2} \hat{P}^{2}(k)}{\partial k^{2}}\left[\frac{\sin{(Gk/2)}}{k/2}\right]^{2}}{\int \mathrm{d}k\, \hat{P}^{2}(k)\left[\frac{\sin{(Gk/2)}}{k/2}\right]^{2}}.   $$

This expresses the MSD as a ratio of two single integrals over reciprocal space. Each integrand is a product of two separate factors whose conceptual sources are unrelated to each other. One factor is the probe function $\phantom {\dot {i}\!}\left [\frac {\sin {(Gk/2)}}{k/2}\right ]^{2}$ while the other, the square of the Fourier transform $\phantom {\dot {i}\!}\hat {P}(k)$ of $\phantom {\dot {i}\!}e^{-U(x-x_{c})/D}$ in the denominator but of the transform of its second *k*−derivative in the numerator, is an animal motion quantity. The former is determined only by the grid size, totally independently of the motion characteristic of the animals. By contrast, the latter is given solely by the animal motion and is independent of the observational probe characteristics, i.e. of the grid size. Importantly, the home range *L* is given by

(10) $$ L = \sqrt{-2\left[\frac{\frac{\partial^{2} \hat{P}(k)}{\partial k^{2}}}{\hat{P}(k)}\right]_{k=0}}.  $$

For uniform *ρ*(*x*_*c*_) Eqs. (9) and (10) can also be written [126], respectively, as

(11) $$ {}{\small{\begin{aligned} \langle \Delta x^{2}\rangle_{\text{ss}}=\frac{G\int_{-G}^{G}\mathrm{d}y\,y^{2}\,f(y)-\left[\int_{0}^{G}\mathrm{d}y\,y^{3}\,f(y)-\int_{-G}^{0} \mathrm{d}y\,y^{3}\,f(y)\right]}{G\int_{-G}^{G}\mathrm{d}y\,f(y)-\left[\int_{0}^{G}\mathrm{d}y\,y\,f(y)-\int_{-G}^{0}\mathrm{d}y\,y\,f(y)\right]}, \end{aligned}}}  $$

where $f(x)=\int _{-\infty }^{+\infty }dz\,\exp \left [-\frac {U(z)+U(z-x)}{D}\right ]$ is the convolution of $\exp [\!-U(x)/D]$ with itself, and

(12) $$ L=\sqrt{\frac{2\int_{0}^{+\infty}\mathrm{d}x\,x^{2}e^{-\frac{U(x)}{D}}}{\int_{0}^{+\infty}\mathrm{d}x\,e^{-\frac{U(x)}{D}}}}.   $$

Extensions of Eq. (11) and (12) in 2*d* are straightforward and allow for observation windows of rectangular shape and for a potential along the orthogonal axis which can be different from *U*(*x*).

**Figure 1:**
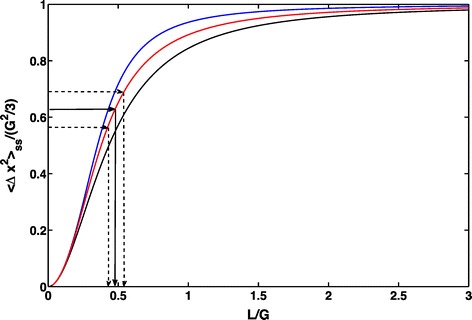
**Graphical representation to infer the average size of an individual home range** ***L*** **from movement data gathered within a square observation window of size** ***G*** ^**2**^ **.** The three sigmoidal curves in red, black and blue are generated by plotting, respectively, the 2*d* analogue Eq. (11) with the potential $U(x)/D=\left \{\text {erf}\left [ \sqrt {6\pi /11}(x/L+1/2)\right ]-\text {erf}\left [ \sqrt {6\pi /11}(x/L-1/2)\right ]\right \}$, Eq. (14) with $U(x)/D=\ln \left (1+2x^{2}/L^{2}\right)^{3}$, and Eq. (13) when *U*(*x*)/*D*=*x* ^2^/*L* ^2^, and their corresponding equivalent along the orthogonal axis.

The practical prescription provided by this analysis works as follows. Consider the ratio *ζ*=*L*/*G* of the unknown to the known length. Equation (11) allows one to plot a curve for the dependence of the MSD on the parameter *ζ*. The curve turns out to be sigmoidal in shape. The known value of the ordinate allows one to read off the value of *ζ*. Since *G* is known, *L* is obtained directly. The disentangling of the two length scales is thus done without any confusion or interference between the space-restriction effects of the two lengths. In Figure 1 we show how different potential shapes result in qualitatively similar dependence of 〈*Δx*^2^〉_ss_ on *ζ*. We have considered there a harmonic potential, a multi-harmonic potential and a logarithmic potential. By the phrase multi-harmonic we mean a potential that has two linear (harmonic) regimes with different strengths close to and far from the den or burrow. We do not display the explicit expression for the multi-harmonic potential because it is cumbersome. For the other two potentials we have, respectively,

(13) $$ {} {\small{\begin{aligned} \frac{\langle \Delta x^{2}\rangle_{\text{ss}}}{G^{2}/6}=6\zeta^{2}\left\{\!1\,+\,\frac{\sinh\left[(1/2\zeta)^{2}\right]}{\sinh\left[(1/2\zeta)^{2}\right]\,-\,\frac{1}{2\zeta}\sqrt{\frac{\pi}{2}}e^{(1/2 \zeta)^{2}}\text{erf}\left(\frac{1}{\sqrt{2}\zeta}\right)}\!\right\}\!, \end{aligned}}}  $$

where $\text {erf}(z)=(2/\sqrt {\pi }){\int _{0}^{z}}\mathrm {d}t\,e^{-t^{2}}$ is the Gauss error function, and

(14) $$ {}\frac{\langle \Delta x^{2}\rangle_{\text{ss}}}{G^{2}/6}=6\left\{\frac{\zeta}{\arctan\left(\frac{1}{2\zeta}\right)-\zeta\ln\left[1+\frac{1}{(2\zeta)^{2}}\right]}-4\zeta^{2}\right\}.   $$

From Eq. (13) and (14) one notices that the parameters *ζ*=*L*/*G* and *G*^2^ completely determine the saturation value of the MSD, which is expected given the presence of only two spatial scales *L* and *G*. To isolate the dependence on *ζ*, it is more convenient to normalise the MSD to the size of the observation domain *G*^2^/6, the factor 6 being present to make the right hand-side of Eqs. (13) and (14) reach 1 for $\zeta \rightarrow +\infty $.

From the knowledge of *G* and the value of 〈*Δ***x**^2^〉_ss_ obtained from movement data, one can then graphically extract the home range extent *L* [109,110]. In Figure 1 we display three horizontal black segments representing the value of 〈*Δ***x**^2^〉_ss_ and its errors obtained from hypothetical movement observations. From the intercepts of these segments with the sigmoidal curve corresponding to the particular selected potential (the red one in this example), one can draw three vertical segments whose intercepts with the horizontal axis yield the value of *L*/*G* and its errors. Following this graphical inversion procedure, Giuggioli *et al.* [109] and Abramson *et al.* [110] have deduced the values of the home ranges of two different kinds of mice, *Zygodontomys brevicauda* in Panama and *Peromyscus maniculatus* in New Mexico, respectively (see more details in Section “Applications to observations in the field”).

Once home ranges are quantitatively measured in the manner explained, important questions arise: how to describe their consequences on animal dynamics, and what measurable effects these consequences have on known phenomena involving the animals. The first question is easily answered in that Smoluchowski equations such as Eq. (7) must be considered. Their propagators, obtained for instance through Ornstein-Uhlenbeck arguments are well-known [66] and yield interesting consequences [128] on the motion of random walkers under confinement. The second question has been answered in a recent investigation of the transmission of infection when animals move under confinement [111]. A surprising result has been found that the existence of finite home ranges can have unexpected effects on the efficiency of the transmission. A change in the diffusion constant or the strength of confinement (the latter being inversely related to *L*) has non-monotonic consequences on infection: an increase in *D* or a decrease in *L* might tend to increase the infection efficiency but only up to a point. Beyond an extremum, the changes have the opposite effect. What this means is that optimum values of *D* or *L* exist, departures from which always cause a decrease in the efficiency of infection transmission. Home range confinement is, thus, a nontrivial characteristic of animals in ecological investigations.

## Mutual interactions leading to territoriality: the effects of time scale disparity

Many-body problems with mutual interactions are always much more difficult to solve, in any field of science, than those involving non-interacting individuals subjected to external fields. The present section deals with this aspect of our study and is consequently the largest in the article. An understanding of the collective feature of territory emergence is the aim. We focus on a particular approach that one of the coauthors of this article has taken along with his collaborators [75], by constructing the so-called territorial random walker (TRW) model, but describe also alternatives that have been proposed earlier [63,64,129-131].

The TRW model bears similarities to the autocatalytic model developed in 1989 by Deneubourg and co-workers [132,133] used to represent foraging Argentine ants that explore new areas following the pheromone trails left by others. A large literature on models of movement whereby individuals choose directions and steps according to the signals present in the environment have also appeared later, and are often referred to as reinforced [134] or active random walkers [135]. Examples include the formation of dendritic foraging patterns by ants [136], aggregation of myxobacetria [137], movement with preferential relocations to places visited in the past, [138] and many others (see e.g. [59]).

The TRW model is a stochastic computational model representing a set of random walkers, the animals, moving on a discrete lattice (with periodic boundary conditions) in continuous time. As an individual moves on the lattice it deposits a mark, which remains active for a finite time $\mathcal {T}_{A}$. Upon the encounter of a foreign mark an individual interacts by retreating in a random direction away from foreign marks. Interestingly these reaction rules do not require any information retrieval on the part of the individuals because recollection of the locations visited by others is held in the environment rather than in the animal. Non-overlapping territories or marked areas are generated at each instant of time by the exclusion dynamics [77] of the TRW model [75].

To reproduce spacing patterns with overlapping marked areas requires changing the mechanism of avoidance interaction from full to partial retreat. This has been shown to be the case in a variation of the original model [53] that accounts for the animal ability to respond differently depending on how long ago a mark was deposited [139]. In its simplest form, i.e. for diffusing individuals, the reaction mechanism is implemented computationally by having, at time *t*, the probability for an animal at location (*m*,*n*), say along the horizontal and vertical axis, to move left, right, up and down, respectively, *l*_+_, *l*_−_, *u*_+_, and *u*_−_, given by

(15) $$ \begin{aligned} l_{\pm}(t,\tau)&=\frac{1}{4}\left\{1\pm\left[2p(\tau)-1\right]\kappa_{l}(m,m_{c},n,n_{c})\right\},\\ u_{\pm}(t,\tau)&=\frac{1}{4}\left\{1\mp\left[2p(\tau)-1\right]\kappa_{u}(m,m_{c},n,n_{c})\right\},  \end{aligned}  $$

where (*m*_*c*_,*n*_*c*_) is the centroid position of an animal marked area at time *t*, $\kappa _{l}(m,m_{c},n,n_{c})=(m-m_{c})/ \sqrt {(m-m_{c})^{2} + (n-n_{c})^{2}}$, $\kappa _{u}(m,m_{c},n,n_{c})=(n-n_{c})/\sqrt {(m-m_{c})^{2} + (n-n_{c})^{2}}$. The the retreat bias, or avoidance response, *p*(*τ*) is a function of the age *τ* of the encountered mark. When *p*=1/2 for all *τ*, the walkers ignore the scent produced by others, whereas the choice *p*(*τ*)=1 for $\tau \leq \mathcal {T}_{A}$, and equal to 1/2 for all other *τ* values, was used in the original TRW model—with random bias away from foreign scent rather than through Eq. (15). It is possible to show that a Master equation in discrete space governed by rates given by Eq. (15) with *p*(*τ*) independent of *τ* reduces to the 2d Holgate-Okubo localising tendency model in the continuum limit [140].

Although many other *p*(*τ*) choices to obtain overlapping marked areas are possible, a convenient functional dependence that satisfies the requirements *p*(0)=1 and $p(\mathcal {T}_{A})=1/2$, is given by the single *α*-family

(16) $$ p(\tau)=\frac{1}{2}\left\{\begin{array}{cc} 1+\sqrt{1-\left(\frac{\tau}{\mathcal{T}_{A}}\right)^{\alpha}} & \qquad \tau\leq \mathcal{T}_{A} \\ 1 & \qquad \tau>\mathcal{T}_{A} \end{array} \right.   $$

**Figure 2:**
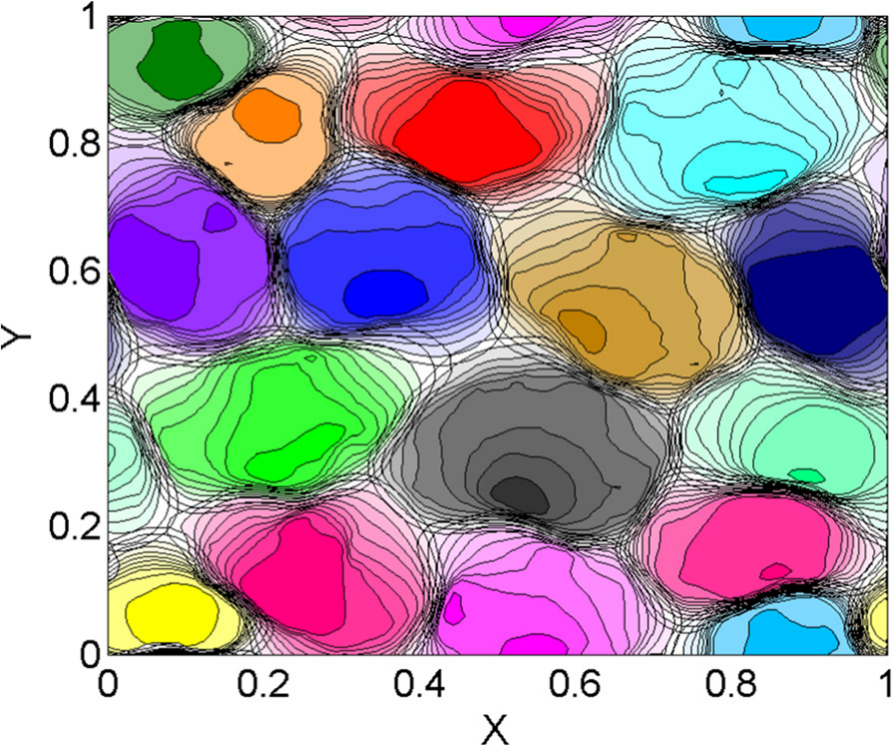
**Utilization distribution from a run of the territorial random walk model with 16 individuals (in a periodic lattice of 25 ×25 sites), a spatial competition strength of** ***Z*** **=32, and with the parameter** ***α*** **that controls the retreat response upon the encounter of foreign marks being 10 **[53]. The emerging home ranges have been reconstructed over a time span equal to 2.5 times the decay time ${\mathcal {T}}_{A}$ of the marks. The contour level values of the utilization distribution from the outer most to the inner most, when all present, are 10 ^−4^ multiplied, respectively, by the following factors: 1, 2, 4, 6, 8, 10, 12, 14, 20, 40, and 80. Simulation code to generate this figure are freely available at doi:10.5061/dryad.v60r7.

Notice that the bias implementation of the walk in Eq. (15) and (16) implies that the individuals possess some degree of spatial memory as well as navigational abilities to be able to determine the centroid location of their own marked area. In Figure 2 we present an example of the emerging territorial pattern with an avoidance response corresponding to *α*=10.

The spatial patterns emerging from the dynamics of conspecific avoidance depend on initial locations and scent mark profiles as well as the specific random realisation of the movement paths. Although these various degrees of stochasticity produce a rich repertoire of shape and size of the resulting home ranges, two important characteristic scales of the territorial random walk model can be identified: the movement rate that defines how quickly an animal covers the available territory and the rate of decay $\mathcal {T}_{A}^{-1}$ of the deposited marks. If an animal succeeds in revisiting past locations before the time $\mathcal {T}_{A}$ has elapsed, it maintains those locations as part of its territory by refreshing the old marks. An animal thus needs to return to a scented location to maintain that location as part of its territory. The mean return time to a system subset can be calculated for discrete stochastic models, using the Kac recurrence lemma [141] as the ratio of all possible configurations of the system divided by the number of configuration of the subset. In our case, for a discrete random walker on a confined 2*d* lattice with *n* sites, the mean return time to a specific site is thus equal to *n*, i.e., the area of the confined space. Taking the average territory size as the inverse of the population density *ρ*, the mean return time is simply obtained by rescaling *ρ*^−1^ with 4*D*, where the multiplicative factor 4 is chosen for convenience so that $4D\mathcal {T}_{A}$ represents the average area explored by a 2d random walker within time *T*_*A*_. The ratio between the time for the marks to remain active and the mean return time (4*Dρ*)^−1^ is thus $Z=4D\rho \mathcal {T}_{A}$.

This parameter *Z* has also an intuitive interpretation in terms of spatial scales [53]: it is the ratio between the average area that a diffusing animal would cover in a time $\mathcal {T}_{A}$ and the average size *ρ*^−1^ that each animal would occupy if the terrain was equally divided into exclusive regions among the individuals of the population. When the probability of retreat upon the encounter of foreign marks is high, from the perspective of a focal individual an increase in $\mathcal {T}_{A}$ makes its marks on the terrain last longer. Similarly an increase in *ρ*, reduces the space available to each animal and causes the focal individual to encounter the edge of its marked area more frequently. As a result *Z* has been named the spatial competition parameter [53] because an increase of either $\mathcal {T}_{A}$ or *ρ* makes territorial marks persist longer, either preventing others from acquiring additional space or limiting the depth of intrusion into foreign territories.

### Replacement of the interaction field with moving walls: a simplified picture

The spatial competition parameter is important for the construction of a simplified picture of the emergence of spacing patterns in the territorial random walk model. Small and large *Z* correspond respectively to fast and slow dynamics of territory boundaries. In the regime of slow territory dynamics, fluctuations of mark locations are limited by their extended persistence and the dynamics of the territories reduce mainly to those of the boundary marks. It is this regime that represents more closely ecological scenarios in which an animal moves relatively quickly within a region whose boundaries are not static but fluctuate over slower time scales [75].

In the regime with strong spatial competition, it is possible to obtain a simplified mathematical description that links the reaction response of the individual animals to the formation of territorial patterns. This simplification is made possible because of the time-scale disparity between the movement rate of the animals and the territory boundaries. Time-scale disparity arguments are commonly employed in interpreting physical problems [142], but they have also been used extensively in the ecological literature, e.g. in studies of intra- and inter-patch dynamics [143,144].

**Figure 3:**
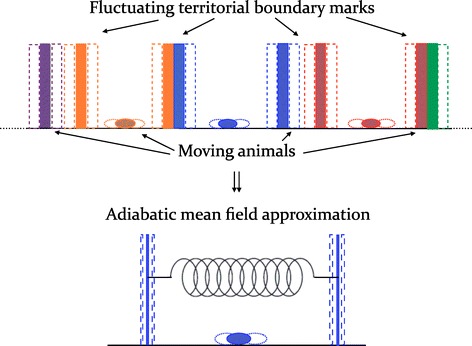
**Pictorial representation of the adiabatic approximation for a 1** ***d*** **case, which allows the reduction of the many-body problem of territorial random walkers to a one-body problem.** In the latter one walker moves within two fluctuating boundaries. Their dynamics follow the exclusion statistics and display a tendency to return to their equilibrium value proportional to the separation distance (spring force).

From the viewpoint of a moving individual, the displacement of the boundaries of the neighbouring territories represents a slow perturbation on the spatial configuration of the focal individual. It is thus possible to exploit this time-scale disparity and perform the so-called adiabatic approximation (see e.g. [145]) whereby certain objects, in this case the territory boundaries, are considered immobile on the movement time scale of other objects, the animals. This adiabatic procedure is used in conjunction with a mean field approximation. It can be employed when a large number of subsystems, in this case the individual animals, are in interaction, and the interaction is not weak so perturbation is impossible to use as an approach. The many subsystem situation can then be reduced to one involving a single subsystem interacting with another single average subsystem representing all the others, in this case the fluctuating boundaries. The spatio-temporal dynamics of many individuals and their boundaries are thereby transformed into those of a focal individual within two slowly fluctuating boundaries; the boundaries and their motion represent, in this mean field manner, the actual complex interactions with the rest of the population. Formally this is accomplished by taking the occupation probability of the animal and the boundaries of its territory as being the product of the occupation probability of the animal, *W*, with that of the boundaries *B*, viz. $P(\vec {x},\vec {L},t)\approx W(\vec {x},t|\vec {L})B(\vec {L},t)$, where $\vec {x}$ and $\vec {L}$ represent the individual’s and boundaries’ location and $W(\vec {x},t|\vec {L})$ is the animal position at time *t* given that the size of the territory is *L*. In Figure 3 we show pictorially the adiabatic and mean field approximation for territorial random walkers in 1*d* for the case when $\alpha \rightarrow +\infty $, that is when the retreat response is certain if the encountered foreign mark is not older than $\mathcal {T}_{A}$.

### Characterising the movement of the animal

The movement statistics of the individual unaffected by the encounter of foreign marks determines the functional dependence of *W*. In continuous space, *W* satisfies the generalised master equation, described in Section “Fundamental considerations for mathematical development”, with the associated boundary conditions at the territory edges. For the case of full territorial exclusion, i.e. when individuals always retreat upon the encounter of foreign marks, the animal occupation probability in Cartesian coordinates is given by the solution of

(17) $$\begin{array}{@{}rcl@{}} &&\frac{\partial{W(x,y,t)}}{\partial t}=D{\int_{0}^{t}}\mathrm{d}s\,\phi(t-s)\left(\frac{\partial^{2}}{\partial x^{2}}+\frac{\partial^{2}}{\partial y^{2}}\right)W(x,y,s),  \\ &&\left.\frac{\partial W(x,y,t)}{\partial x}\right|_{x=L_{1x},L_{2x}}=\left.\frac{\partial W(x,y,t)}{\partial y}\right|_{y=L_{1y},L_{2y}}=0.  \end{array} $$

The second line of Eq. (17) represents the no-flux boundary conditions indicating that an animal cannot escape from its territory with *L*_1*x*_ and *L*_2*x*_, respectively, the leftmost and rightmost territory edge along the *x*-axis, and *L*_1*y*_ and *L*_2*y*_ along the *y*-axis. The so-called memory *ϕ*(*t*) characterises the degree of correlation or anti-correlation of the animal steps. For the case of a persistent walk, the choice *ϕ*(*t*)=(*v*^2^/*D*)*e*^−*t*/*T*^ represents an animal that moves at speed *v* in the same direction without turning for an average time *T*. In this case, the solution of Eq. (17) is separable along each axis and reduces to the product $W(\vec {x},t)=\mathcal {W}_{x}(x,t)\mathcal {W}_{y}(y,t)$, whose mathematical expressions can be found in [146].

**Figure 4:**
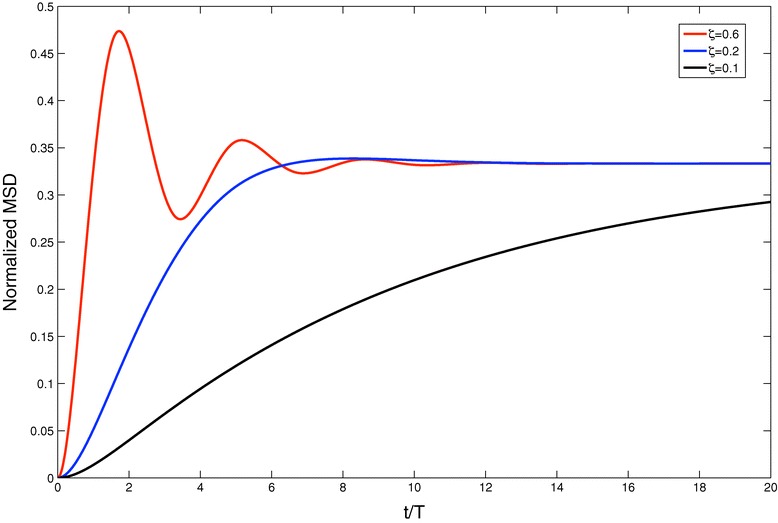
**MSD plot as a function of** ***t*** **/** ***T*** **for a square territory normalised to the size of the confining area for different values of the relative correlation** ***ζ*** **.** In this case the territory boundaries are fixed.

To obtain analytic expressions for the MSD, one needs to multiply Eq. (17) by $(\vec {x}-\vec {x}_{0})^{2}$ and integrate over all possible *x* and *y* coordinates within the domain (see Eq. (C1) in [146]). Further integration over all possible initial conditions gives the MSD expression

(18) $$ {\small{\begin{aligned} \langle (\vec{x}-\vec{x}_{0})^{2} \rangle(t)=&{\lambda_{x}^{2}}\left\{\frac{1}{6}-\frac{16}{\pi^{4}}\sum_{n=1}^{+\infty}\frac{1}{(2n-1)^{4}} \left[\vphantom{\frac{\sin(\Theta_{n,x}t)}{2T\Theta_{n,x}}}\cos\left(\Theta_{n,x}t\right)\right.\right.\\&\quad+\left.\left.\frac{\sin(\Theta_{n,x}t)}{2T\Theta_{n,x}}\right]e^{-\frac{t}{2T}}\right\}+\\ &{\lambda_{y}^{2}}\left\{\frac{1}{6}-\frac{16}{\pi^{4}}\sum_{n=1}^{+\infty}\frac{1}{(2n-1)^{4}} \left[\vphantom{\frac{\sin(\Theta_{n,y}t)}{2T\Theta_{n,y}}}\cos\left(\Theta_{n,y}t\right)\right.\right.\\&\quad+\left.\left.\frac{\sin(\Theta_{n,y}t)}{2T\Theta_{n,y}}\right]e^{-\frac{t}{2T}}\right\}, \end{aligned}}}   $$

where $\Theta _{n,z}=(2T)^{-1}\sqrt {4n^{2} \pi ^{2}{\zeta _{z}^{2}}-1}$ with *ζ*_*z*_=*vT*/*λ*_*z*_, which is precisely *L*_2*z*_−*L*_1*z*_, i.e. the position of the right boundary minus that of the left boundary along each axis. The presence of cosine and sine, rather than purely exponential terms, in the time-dependence of $\langle (\vec {x}-\vec {x}_{0})^{2} \rangle (t)$ may give rise to oscillations at intermediate times, which are displayed in Figure 4 for the case of a square territory (*λ*_*x*_=*λ*_*y*_). An analysis of the expression (18) shows that oscillations in the MSD oscillations appear whenever *ζ*_*z*_>(2*π*)^−1^, which corresponds to situations when *Θ*_1,*z*_ is a real number. Expressions for the probability distribution and the MSD for the case of a fixed territory with circular shape can also be constructed and can be found in [146].

### Describing the movement of the boundaries

Having specified $W(\vec {x},t|\vec {L})$, we now turn to the time dependence of the probability distribution of the boundary positions $B(\vec {L},t)$ which biologically represents two competing effects: the acquisition of new territory by the resident animal and the pressure of the neighbours for territorial takeover. In the adiabatic regime, a mean field prescription to describe the displacement of the boundaries is to ignore the detailed dynamics of the neighbours and their marked areas, accounting only for the slow movement statistics of the boundaries. As the boundary locations have a tendency to move and reduce (increase) the territory size when larger (smaller) than the equilibrium value, which is the inverse of the population density, a useful approximation is to impose that the boundaries are forced by an assumed spring that maintains the equilibrium territory size.

To formally describe both the sub-diffusive dynamics of the boundaries and their return tendency towards the equilibrium size, a Fokker-Planck equation with a time-dependent diffusion constant *φ*(*t*) [147] has been employed. Along the formalistic lines of [73,148], one can write

(19) $${\kern1pt} \begin{aligned} \frac{\partial B(\vec{L},t)}{\partial t}&= K\varphi(t)\left\{\nabla^{2}+\frac{\gamma}{2}\sum_{z}\left(\frac{\partial }{\partial L_{2z}}-\frac{\partial }{\partial L_{1z}}\right)\right.\\ &\qquad\;\;\times\left.\left[L_{2z}-L_{1z}-\bar{L}_{z}\right]\vphantom{\nabla^{2}+\frac{\gamma}{2}\sum_{z}\left(\frac{\partial }{\partial L_{2z}}-\frac{\partial }{\partial L_{1z}}\right)}\right\}B(\vec{L},t), \end{aligned}   $$

where *K* is the boundary diffusion constant, ∇^2^ is the Laplacian operator in the Cartesian coordinates $\vec {L}=(L_{1x},L_{2x},L_{1y},L_{2y})$, *γ* is the phenomenological spring constant expressed in units of inverse length, and $\bar {L}_{z}$ is the average territory length along each trajectory ($\bar {L}_{x}\bar {L}_{y}$ is equal to the inverse of the population density). Notice that in Eq. (19) the interaction term $\mathcal {U}(\vec {L})$, described in general terms in Eq. (4), is of the pairwise form $\mathcal {U}(L_{2z},L_{1z})=L_{2z}-L_{1z}-\bar {L}_{z}$ and $\mathcal {U}(L_{1z},L_{2z})=-(L_{2z}-L_{1z}-\bar {L}_{z})$—as discussed in the introduction rare are the situations where interaction is considered to occur as resulting from non-pairwise events. These interaction terms in Eq. (19) represent the derivative of a harmonic potential centered around $\bar {L}_{z}$ in both directions and for that reason there is a summation along the two axes. When *φ*(*t*) is time-independent, ${\int _{0}^{t}}\mathrm {d}s\,\varphi (s)$ is linear in *t*, and Eq. (19) reduces to a diffusive case. On the other hand, choices with sub-linear ${\int _{0}^{t}}\mathrm {d}s\,\varphi (s)$ reproduce the sub-diffusive scaling in the MSD observed in the full-blown stochastic simulations of territorial random walkers. In 1*d*, the boundary MSD scales as $\sqrt {t}$ [76], a characteristic feature of tagged particle dynamics in single file systems (see e.g. [84]). In a 2*d* lattice, stochastic simulations for intermediate times display logarithmic corrections proportional to $t/\ln (t)$ [75], which have been used to represent the long-time dependence of the MSD.

The solution of Eq. (19), supplemented by the boundary condition that prevents the left and right boundary along each axis to exchange order, can be obtained by variable separation in the centroid and separation distance [76]. The resulting probability distribution for each axis is given by the product $\mathcal {R}_{z}(\lambda _{z},t)Q_{z}(\mathcal {L}_{z},t)$, with  controlling the dynamics of the boundary edge separation and  that of the boundary centroid. The computation of the MSD in this case shows that the long-time dependence is controlled only by ${\int ^{t}_{0}}\mathrm {d}s\,\varphi (s)$, which results from the exclusion statistics of the territory boundary. This long time dependence provides the means to link quantitatively the outcome of the stochastic simulations with the microscopic mechanism of mark avoidance, in particular the relation between the value $\mathcal {T}_{A}$ during which deposited marks remain active and the diffusion constant *K* (see Section “Applications to observations in the field”).

### Comparison to an earlier approach

The model by Lewis, Moorcroft and Murray (LMM), successfully applied to movement data on wolves [63] and coyotes [149], represents the avoidance interaction between animal pairs by coupling the animal occupation probability of one individual to the distribution profile of the scent of the other. As a result, the movement bias (retreat) in the LMM model is due to a spatially extended interaction potential as compared to sharply peaked walls implemented in the TRW model.

The scent deposition is implemented differently in the two models: with a constant rate independent of the motion in the LMM model, and at regularly spaced intervals whenever an animal moves in the TRW model. This implies that the former model is more suited to model animals that leave consecutive marks further apart as they move quicker over the terrain, whereas the latter model is more suited to model animals that leave no gaps in the terrain between marks. Furthermore, over-marking (increase in marking rate when animals encounter foreign scent) is present so far only in the LMM model [150].

In the LMM model the presence of an attractive potential towards the den site [151] forces the animal occupation probability to reach a steady state. The TRW model, on the other hand, does not possess a steady state. Fluctuations in the boundary locations are always present, except for extremely large values of the active scent time. A comparison of the models in this regard is therefore not straightforward. However, the addition of a bias towards a burrow in the TRW model, e.g. to represent animals that display site fidelity [152], destroys the dynamic nature of the TRW model forcing the occupation probability of each individual to reach a steady state. One can then compare this steady state profile when no over-marking occurs in the LMM model. A further discussion can be found in [140].

In summary, although a microscopic description of the movement of the animals is present in both models, a representation of the discreteness of the events [153] of (avoidance) interaction are present only in the TRW model [153]. In the latter, tracking when and where scent is deposited allows one to define a territory as the set of locations visited by an individual within the time over which animals respond to the encounter of foreign scent. The difficulties in identifying the ever-changing locations of territory boundaries are thereby eliminated. The TRW model thus provides the long-sought operational definition of a territory in scent-marking animals.

## Applications to observations in the field

Although the estimation of movement patterns from recordings of animal locations has a long history (see e.g. [96,154]), recent years have seen an explosion in the number and quality of field observations [62] due to the rapid development of cheap and easy to use tracking sensors and loggers. A rich platform has thereby been provided for empiricists and theoreticians to help each other answer fundamental questions in animal behaviour [155]. Inspired by the original studies on diffusive and persistent processes [156-159], various approaches that aim at extracting movement features and environmental drivers have emerged: change-point analysis [160], Brownian bridges [161,162], Hidden Markov models and state-space models [163-166], and others such as the partial sum approach [167]. A common feature of these studies is the ability to account for the spatial and temporal heterogeneity in the observations.

These heterogeneities are also of concern in studies on animal home ranges and territories since they affect the patterns of space use [36]. Movement attributes are more difficult to extract when animals roam in confined space. Part of this difficulty is associated with the fact that home range and territory boundaries are not insurmountable barriers for the animal.

For central place foragers, an approach that characterises an animal space use pattern, avoiding the precise demarcation of the confining boundaries, consists of representing the tendency of an individual to return to its burrow or den site through a phenomenological attraction force [151]. The functional dependence of the force defines the spatial dependence of the drift towards the den site. If the animals diffuse with a diffusion constant *D* and with a drifting force **F** such that $\textbf {F}=-\frac {\partial }{\partial x}U(x-x_{c})\textbf {i}-\frac {\partial }{\partial y}V(y-y_{c})\textbf {j}$ with **i** and **j** the unit vector along two orthogonal axes *x* and *y*, respectively, the spatial dependence of the probability distribution *P* at long times reduces to

(20) $${} {\fontsize{9.4pt}{9.6pt}\selectfont{\begin{aligned} P(x,y,t\rightarrow+\infty)=\frac{e^{-\left[U(x-x_{c})+V(y-y_{c})\right]/D}}{\int_{-\infty}^{+\infty} \mathrm{d} x\,e^{-U(x-x_{c})/D}\int_{-\infty}^{+\infty}\mathrm{d}y\, e^{-V(y-y_{c})/D}} \end{aligned}}}   $$

where the potential *U* and *V* have a minimum, respectively, at *x*_*c*_ and *y*_*c*_, i.e. the location of the den site. As this procedure relies upon reconstructing the long-time occupation probability of an individual, it requires independent observations of an animal’s positions. When animal fixes are gathered with sufficiently small sampling rate, the movement data can be fitted to reconstruct the spatial dependence of the animal occupation probability with a fit to Eq. (20) [109,110].

Although a parametric fit to $P(x,y,t\rightarrow +\infty)$ may give a good estimate of the functional dependence of $\left [U(x-x_{c})+V(y-y_{c})\right ]/D$, it is necessary to obtain an independent measurement of *D*. In other words, to disentangle the randomness of the movement from the determinism inherent in the animal drift towards the den site, it is necessary to quantify the stochasticity of the animal trajectories. To perform this task, a useful quantity to identify the statistical features of a movement process from recordings of animal locations is the time dependence of the squared displacement. It has been employed in many contexts including, for instance, for the study of exciton transport in organic crystals [168,169]. More recently it has become the subject of a variety of investigations in animal movement studies [71,170-174] since it provides a synthetic measure of the stochastic features of the individual trajectories. By averaging multiple observations of different individuals [109,110] or by performing a time-window average (see e.g. [175,176]) over a single animal trajectory when tracking occurs over a sufficiently long time, information about the MSD can be obtained.

### Aggregate measurements from multiple trajectories

Studying the time-dependence of the MSD at sufficiently short time, such that the animal displacements are not affected by the home range boundaries, also allows the detection of non-diffusive features of the trajectories when present. By considering a general time-dependence of the form 〈*Δ***x**^2^〉∼*t*^2*H*^ where *Δ***x** indicates the displacement in 2*d* of an individual from the initial location **x**_0_ and *H* is the so-called Hurst exponent [177], one can extract the anomalous exponent 2*H* and its associated fractal dimension *δ* of the animal trajectory through the relation *δ*=2−*H* [178]. It is possible to do that by extending the analytical methods presented in Section “Interactions with the environment: the emergence of home ranges” to compute the *q*-th moment of an animal occupation probability.

Extracting a single parameter *H* to represent the statistical features of the movement process may be insufficient to account for the nuances of the experimental observations. In such cases it becomes necessary to analyze the *q*-th moment of the occupation probability, which coincides with the MSD when *q*=2. A trajectory is said to be multifractal, rather than monofractal, when the Hurst exponent is *q*−dependent, that is 〈|*Δ***x**|^*q*^〉(*t*)∼*t*^*qH*(*q*)^ rather than 〈|*Δ***x**|^*q*^〉(*t*)∼ [ 〈*Δ***x**^2^〉(*t*)]^*q*/2^∼*t*^*qH*^. By taking the logarithm of the ratio of the *q*-th moment at time *t*_1_ and subsequently at time *t*_2_, one can use the exact relation [147]

(21) $$ H(q)=\ln\left[\frac{\langle|\Delta \mathbf{x}|^{q}\rangle(t_{1})}{\langle|\Delta \mathbf{x}|^{q}\rangle(t_{2})}\right]\frac{1}{q\ln(t_{1}/t_{2})}   $$

to determine the multifractal nature of the trajectory. In its simplest form, the *q*-th moment is obtained by aggregating the data of individuals of the same population moving within their own home range, which means extracting from the experiment the equivalent of

(22) $$ {} \langle |\Delta \mathbf{x}|^{q}\rangle(t)=\frac{\int\int \mathrm{d}^{2}x_{0}\int\int \mathrm{d}^{2}x\,|\mathbf{x}-\mathbf{x}_{0}|^{q} \mathcal{P}_{\mathbf{x}_{0}}(\mathbf{x},t)\mathcal{I}(\mathbf{x}_{0})}{\int\int \mathrm{d}^{2}x_{0}\int\int \mathrm{d}^{2}x\,\mathcal{P}_{\mathbf{x}_{0}}(\mathbf{x},t)\mathcal{I}(\mathbf{x}_{0}) }.   $$

Here $\mathcal {P}_{\textbf {x}_{0}}\!(\textbf {x},t)$ represents the time-dependent solution of

(23) $${} \begin{aligned} \frac{\partial P(x,y,t)}{\partial t}&=D(t)\left\{\frac{\partial}{\partial x}\left[\frac{\partial }{\partial x}+\frac{\mathrm{d}U(x-x_{c})}{\mathrm{d}x}\right]\right.\\&\qquad\quad\;+\left.\frac{\partial }{\partial y}\left[\frac{\partial }{\partial y}+\frac{\mathrm{d}V(y-y_{c})}{\mathrm{d}y}\right]\right\}P(x,y,t), \end{aligned}   $$

and $\mathcal {I}(\textbf {x})=\mathcal {P}_{\textbf {x}_{0}}(\textbf {x},0)$. The convenience of Eq. (23) lies in its flexibility to capture anomalous statistical features of the animal walk through *D*(*t*) and the expected steady state solution (20).

The effects of spatially limited observations on the estimation of home range size mentioned already in Section “Interactions with the environment: the emergence of home ranges” also apply here when estimating the Hurst exponent *H*(*q*) with the integration limits in Eq. (22) becoming finite. The moments with high *q* are heavily affected by the presence of a limited sampled area as they contain spatial information about the tail of the probability distribution. In certain cases, e.g. with square observation windows and complete uncertainty about the initial position of the individuals, i.e. when $\mathcal {I}(\textbf {x}_{0})$ is uniform, the integrals can be computed explicitly. A simple expression for *H*(*q*) then emerges. An application of this analytic procedure to mark-recapture experiments was carried out [147] with a population of *Peromiscus maniculatus* in New Mexico indicating a high degree of correlation in the displacement of the individuals approaching the ballistic limit, possibly due to habitual movement within their home ranges along well defined paths to reduce predation risks.

While the short-time dependence of 〈|*Δ***x**|^*q*^〉(*t*) allows the characterisation of the statistical features of the movement, the long-time dependence gives information about the size of the home range. In an animal population with limited variability in the size of individual home ranges and knowledge about their locations, the MSD expression (22) at $t\rightarrow +\infty $ provides information about home range size. If no information about home range locations is available, one proceeds as in Section “Interactions with the environment: the emergence of home ranges” using Eq. (8).

To determine the shape of the potential *U*(*x*) and *V*(*y*) to be used in Eq. (8), one starts as follows. A histogram of the animal displacements at small and regular time intervals provides a good approximation to the short-time occupation probability of the animals. Selection of the shape and type of potential is done on the basis of observational clues obtained in this manner. Once a potential is selected, a plot of the analytic expression (8) and the subsequent graphical inversion described in Section “Interactions with the environment: the emergence of home ranges” gives the home range size. Following this procedure, mark-recapture data obtained from square grids and web grids of Sherman traps, respectively, of *Zygodontomys brevicauda* in Panama and *Peromyscus maniculatus* in New Mexico were analysed in [109] and [110]. The result of the analysis is summarised in Table 1.

**Table 1:** **Home range and diffusion constant parameters extracted from mark-recapture observations**

**Animal species**	**Geographic region**	
*Zygodontomys brevicauda*	Azuero Peninsula, Panama	
*Peromyscus maniculatus*	New Mexico, USA	
**Potential shape**	**Home range**	**Diffusion constant**
	**length (m)**	**(m** ^***2***^ **/day)**
Box shape	70${\!~\!}^{+50}_{-20}$	200 ± 50
Parabola	100 ± 25	475 ± 50

### Measurements from single trajectories

There has been a realisation in recent times that heterogeneities in the characteristics of the individuals may give rise to spurious interpretation of anomalous movement [179-181]. This has accelerated the development of tools that extract statistical features from individual trajectories, e.g. wavelet analysis [182,183] For animals moving in unbounded domains, recent approaches include the mean-maximal excursion method for subdiffusive processes [184] or the use of Brownian functional maximum likelihood estimators [185,186] for accurate quantification of the diffusion constant for Brownian processes.

For movement in confined space, a promising approach is the one developed to characterize animal movement in circular arenas [187]. With the help of extensive stochastic simulations, it is possible to construct an approximate analytic expression for the MSD of the movement of a (positively) correlated random walker in confined space. Fit to observations provides an effective persistence $\xi =-\mathcal {L}/[\!R\ln (\langle \cos (\theta)\rangle)]$ of the animal, where $\langle \cos (\theta)\rangle $ is the mean of the turning angle distribution in the absence of any reflecting barrier,  is the mean of the step length distribution and *R* is the radius of the arena. The persistence reduces to *ξ*=0 with a uniform turning angle distribution, which corresponds to Brownian motion, progressively increasing as the distribution becomes more peaked around zero. The ballistic motion limit is reached when $\xi \rightarrow +\infty $.

Application of this procedure to a laboratory experiment with rats searching for food pellets appearing at random locations has shown that individual animals move with a directional persistence that minimises the coverage time, i.e. the average time it takes to visit the entire arena [187]. Given its generality and the easy applicability resulting from the use of analytic expressions, this methodology promises to be a useful benchmark to study and interpret foraging processes within home ranges.

### Characterization of movement and active scent-time in territorial animals

Although studies to characterise the movement processes in scent-marking territorial animals abound, only a very small number attempt to extract, simultaneously, information about movement and interaction of the individuals. This is the case of the TRW model presented in Section “Mutual interactions leading to territoriality: the effects of time scale disparity” and applied to location data of red foxes (*Vulpes vulpes*) [188]. Movement data can be fitted to an approximate analytic expression for *P*(*x*,*y*,*t*|*v*,*T*,*γ*,*K*,*L*), the occupation probability for the animal to be at coordinates (*x*,*y*) relative to its home range center at time *t*. The parameters *v*, *T*, *γ*, *K*, and *L*, represent, respectively, the average animal speed, the average time an animal moves before turning, the average rate for a territory size to relax toward the inverse of the population density, the territory border diffusion constant, and the average territory width.

**Figure 5:**
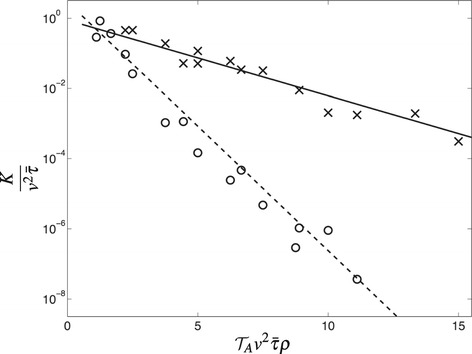
**Relation between territory border diffusion constant** ***K*** **and the dimensionless quantity** $\boldsymbol{\mathcal {T}}_{A}v^{2}\bar {\tau }\rho $ **.** For the diffusive case, the latter is proportional to the competition parameter *Z* described earlier in Section “Mutual interactions leading to territoriality: the effects of time scale disparity”. The data points are the simulation output of the territorial random walk model with full exclusion in the diffusive limit (crosses) and in the ballistic limit (circles). The solid line represents a best fit for diffusing animals, whereas the dashed line is the best fit for animals moving ballistically. Reproduced with permission from [188].

When recordings of the same individual can be considered independent, e.g. when displacement observations occur with sufficiently large intervals of time, it is possible to use the likelihood function [189] $\Xi (v,T,\gamma,K,L)=\sum _{n}\ln [\!p(x_{n}, y_{n}, t_{n}|v,T,\gamma,K,L)]$ where *n* represent the number of location data used. Through the use of an efficient algorithm, e.g. the Nelder-Mead simplex algorithm [190,191], the maximum of the likelihood function gives the best estimate for the mean of each of the five parameters. Resampling the data, e.g. with a bootstrapping algorithm [192], provides the error bars. To extract the value of the active scent-time $\mathcal {T}_{A}$, one resorts to the output of the full-blown stochastic simulations of the territorial random walk model where a linear relation between *K* and the active scent time $\mathcal {T}_{A}$ is established [188]. In Figure 5 we display such a relation. On the vertical axis the territory border diffusion constant divided by $v^{2}\bar {\tau }$ with $\bar {\tau }$ representing the time it takes to move between two lattice sites, that is the ratio between the lattice spacing and the speed *v*. The use of the normalisation factor $v^{2}\bar {\tau }$ allows the comparison of animals moving with different statistics from diffusive to correlated to ballistic.

The exponential dependence of the territory border diffusion constant as a function of the active scent time displayed in Figure 5 can be understood with a simple reasoning based on a first-passage calculation in 1*d*. Focusing on an animal starting at a boundary location *x*_0_, say the right one, in a territory of size equal to *L*, the probability for the territory boundaries not to move requires the animal to move from *x*_0_ to the left boundary and return to *x*_0_ by time $t=\mathcal {T}_{A}$. The diffusion constant of the territory border is thus proportional to the probability for the animal not having moved between its edges. Then $K\propto 1- \int _{0}^{\mathcal {T}_{A}}\mathrm {d}s\mathcal {F}(s)$, where $\mathcal {F}(t)$ is the first-passage probability to start at *x*_0_ and reach the left boundary, and subsequently return from the left boundary to *x*_0_. The first-passage probability from *x*_0_ to the left boundary, and similarly its return to *x*_0_ from the left boundary, is proportional to $e^{-\pi ^{2} D t/4L^{2}}$ [193] where *D* is the diffusion constant of the animal. From this, one realises that $K\propto e^{-\pi ^{2} D\mathcal {T}_{A} /4L^{2}}$. An exponential dependence on $\mathcal {T}_{A}$ thus results.

## Conclusions

The study of animal dynamics and animal interactions is an open subject teeming with activity. Much remains to be understood and much is being done. Below we mention some avenues along which we expect, at least hope, progress to occur in the near future.

An important aspect, not considered here, has to do with the effects of environmental spatial heterogeneities on animal movement and interaction processes. Whereas we have focused in the present article on what we have termed dynamic disorder, such heterogeneities correspond to *static* disorder. The home range models presented in Section “Interactions with the environment: the emergence of home ranges” possess some ability to include spatial heterogeneities not only through the shape of the confining potential, but also through the distribution of home range centres. Better choices for this distribution can be obtained from more detailed landscape models present in the literature, e.g. [42,43]. Introduction of landscape features has been attempted by linking population spatial distribution to animal spatial memory and landscape persistence [194], as well as to prey distribution and terrain steepness [129,149]. All these approaches, however, lack one fundamental aspect, the coupling of the dynamics of the environment with that of the movement and interaction of the individuals. When the spatial heterogeneity is due to a distribution of resources that gets depleted, a detailed study of the resource-animal system becomes necessary.

Inclusion of resource dynamics on the territorial random walk model might be key to answer many of the unresolved issues on the dependence of territoriality and food availability in scent-marking species. Despite the general hypothesis of the inverted U-shaped relationship between territorial behaviour and food availability [195], an interesting long-term study on the Iberian lynx [196] showed that territorial behaviour was unaffected by prey abundance (wild rabbit). Apparently the unpredictability of rabbit abundance makes it more convenient for lynx to maintain exclusive core areas (territories) limiting the number of contacts with other conspecifics [196]. Such behavioural patterns are also seen for other carnivores [197,198]. A detailed modelling of lynx foraging behaviour in a territorial random walk model might provide a mechanistic explanation linking contact rates to search strategies.

Another aspect not included in the approaches that we have described is a formalism capable of accounting for learning abilities and spatial memory. Attempts along this line have been made by Stamps and Krishnan [199,200]. They have analysed spatially implicit models in which individuals learn about competitive abilities of the neighbouring animals via past successes or failures in agonistic encounters [8]. Incorporating this type of learning in the spatially explicit models presented here promises to be a fruitful direction to test ideas on spatial memory [201]. Spatial games of cooperation and detection have also been well studied [202] and could be imported in more detailed representation of avoidance dynamics.

More generally, cognitive processes represent a research avenue with distinct potential. These processes clearly have a role in animal foraging and the formation of movement patterns. Memory, and learned or evolutionarily acquired expectations about landscape attributes, are used by animals to infer the current state of areas not previously visited. It is believed that this is done on the basis of information remembered from previous visits to neighbouring locations [203]. Recollection of a set of favourable or more profitable locations in the habitat has also been shown to be sufficient. Work has been done on models of home range formation in which a single individual displays both an avoidance response to recently visited resource patches, and an attractive response toward resource patches that have been visited sometime in the past [204]. These and other features of the cognitive skills of an animal have only just started to be incorporated in mechanistic models of movement (see e.g. [205]). It is our expectation that they will acquire a prominent role as home range and territory models begin to treat in detail energy costs of locomotion and foraging strategy.

Advances in this direction are expected with improved representation of animal decision-making. An animal searching for food would in fact make decisions based not only on its internal state and sensory inputs, but also on past knowledge and experience, and possible future outcomes. This implies that speed and direction of movement change continuously depending on past, present and expected circumstances. Realistic representation of these decision making processes in a population of interacting animals might hold the key for an improved understanding of the emergence spacing patterns.

Exploration of new regions not visited previously and exploitation of regions already familiar from earlier visits point in different directions. Accordingly, there are tradeoffs responsible for at least two distinct types of territorial patroling observed in scent-marking species. A *hinterland* strategy [206], modelled in Section “Mutual interactions leading to territoriality: the effects of time scale disparityMutual interactions leading to territoriality: the effects of time scale disparity”, ensures that various locations inside the territory are scented regularly, whereas a *borderland* strategy [207] consists of depositing marks only on the outer boundaries of a territory. Examples of the former can be found in red foxes [208], otters [209] and pine martens [210], whereas those of the latter occur in spotted hyaenas [211], meerkats [212] and badgers [213]. To support the idea that sparsely distributed resources may favour a hinterland strategy [214], one should attempt modifications of the original territorial random walk model that include foraging costs and active border patroling, the latter partially explored already in [75]. We hope that mathematical developments already available in other areas will be used for these issues. Examples are general studies on first-passage problems in confined domains [215], and specific studies on partial confinement [216] and escape problems in cellular domains [217].

Red foxes have provided an example of terrestrial animals in the analysis given in Section “Applications to observations in the field”. Although other vertebrate species, such as wolves [218], squirrels [219], and deer [220], are a testbed for the ideas and predictions on territorial defense presented in that analysis, invertebrate species could also exhibit related behaviour. We believe that well studied marine gastropods that exhibit territorial responses are worth exploring to verify certain predictions or to generate novel and unexplored hypotheses on animal spacing. The complex behaviour of the owl limpet *Lottia gigantea* [221,222], a marine gastropod mollusc, appears particularly suitable because contacts with other conspecifics result in avoidance behaviour [223]. As the decision to fight or flee is strongly influenced by recent agonistic successes or failures [224], *L. gigantea* would present an ideal candidate to study how past experiences affect spacing patterns.

The ability to detect mucus of other individuals and its use for territorial marking, as observed in other species [225], could also be explored. The small territory size and slow movements [226] allow one to conduct ecological experiments on marking strategies in a laboratory environment. This permits the identification of whether, and where, individuals interact. The relative ease of manipulation of the food sources and the substrate over which the mollusc moves suggests *L. gigantea* as a model system. With its help, one could study the effects of the environment and intruder pressure on the choices that animals make between maintaining strong social ties and sharing space with the neighbouring individuals [53,227]. The effects of conspecific interactions and resource abundance on this dichotomy has already been observed both in the field in the African golden-wing sunbird [228] and in laboratory experiments in pygmy sunfish [229].

Other examples of invertebrates to investigate, with focus on avoidance mechanisms, are several species in the taxonomic order Diptera, e.g. flies, mosquito and midges. In swarms, while these insects remain globally bound together within a certain volume around a physical marker, local interactions also occur as individuals appear to correlate their displacements with some of their neighbours. Evidence in that direction has been collected from swarming mosquitos, e.g. *Anopheles gambiae* [230] and different species of midges, e.g. *Dasyhelea flavifrons* and *Cladotanytarsus atridorsum* [231], and *Chironomus riparius* [232]. Although these insects move freely throughout the available volume, they form groups without apparent polarisation. Ideas on some form of short time alignment based on velocity and exposure angles of nearby individuals [233,234] together with a mechanism of avoidance of locations recently visited by other individuals may help to explain why despite the lack of collective order, insect swarms are strongly correlated over large length scales. The avoidance here clearly would not rely on scenting the space, since the air does not retain a memory of the passage of these insects. However, past locations visited by other individuals may be retained in the memory of each animal providing a mechanism of exclusion analogous to the one presented for scent-marking species. Testing these ideas of memory-induced avoidance should help develop territorial formation in other non-scenting mammals.

The coupling of animal confinement—man-made, e.g. enclosing fences, or inherent in the animal socioecology—with various types of interactions among the animals, attractive or repulsive, results in profound effects on the transmission of infection in the context of various diseases. As infection is transmitted upon contact or proximity between individuals, the degree of social cohesion of the population determines the direct or indirect animal encounter rate and ultimately the speed of spread of a pathogen. Contact events and disease transmission rates are thus fundamentally linked to the way animals move and respond to their neighbours. Control or management interventions to reduce the prevalence of an infection may become ineffective if the social structure of the animal population is being heavily disrupted, e.g. by culling procedures [235] or by a natural disease [188].

Modelling disease transmission between individuals segregated in different regions of space requires the relaxation of traditional assumptions of homogeneity and well-mixing and demands new theoretical tools capable of treating together the movement and interactions of the animals. While some work has appeared earlier [236,237], a powerful new technique that applies to any number of dimensions, has built-in confinement analysis wherever needed, predicts unexpected insights into epidemic spread, and is suited to the unification of model calculations (for low population densities), and kinetic approaches (for high population densities), has appeared recently [111,112]. We give only a skeletal description.

The concept behind the recent Kenkre-Sugaya treatment of infection transmission is to perform an exact model calculation for low animal densities by treating a single pair of animals represented as tethered random walkers moving diffusively on the terrain, extracting an infection rate in ways formally reminiscent of the Fermi Golden Rule prescription in quantum systems [238], and using the rate in a kinetic equation framework valid for denser systems. The model calculation is based on an early treatment of interacting walkers [239] combined with the Smoluchowski equation description for confinement [128] produced by home ranges. The calculation results in some expected consequences but in some surprising phenomena as well, and yields an infection rate determined by initial conditions as well as the system dynamics. The rate is then fed into a kinetic equation framework similar to that of [99] but augmented to include confinement due to home ranges [105,107,112]. A comprehensive theoretical scheme is thus available and work is in progress both for further development of the scheme and for its application to zoonotic diseases of various kinds including bovine tuberculosis and plague.
